# Comparisons of two diaphragm ultrasound-teaching programs: a multicenter randomized controlled educational study

**DOI:** 10.1186/s13089-019-0137-4

**Published:** 2019-10-03

**Authors:** Eugenio Garofalo, Andrea Bruni, Corrado Pelaia, Giovanni Landoni, Alberto Zangrillo, Massimo Antonelli, Giorgio Conti, Daniele Guerino Biasucci, Giovanna Mercurio, Andrea Cortegiani, Antonino Giarratano, Luigi Vetrugno, Tiziana Bove, Francesco Forfori, Francesco Corradi, Rosanna Vaschetto, Gianmaria Cammarota, Marinella Astuto, Paolo Murabito, Valentina Bellini, Massimo Zambon, Federico Longhini, Paolo Navalesi, Elena Bignami

**Affiliations:** 1 Intensive Care Unit, Department of Medical and Surgical Sciences, University Hospital Mater Domini, Magna Graecia University, Viale Europa-Loc, Germaneto, 88100 Catanzaro, Italy; 2 IRCCS San Raffaele Scientific Institute and Vita-Salute San Raffaele University, Milan, Italy; 3 Department of Anesthesiology and Intensive Care Medicine, Fondazione Policlinico Universitario A. Gemelli IRCCS, Università Cattolica del Sacro Cuore, Rome, Italy; 4 Department of Surgical, Oncological and Oral Science (Di.Chir.On.S.), Section of Anesthesia, Analgesia, Intensive Care and Emergency, Policlinico Paolo Giaccone, University of Palermo, Palermo, Italy; 5 Department of Anaesthesia and Intensive Care, University of Udine, Udine, Italy; 6 Department of Surgical, Medical and Molecular Pathology and Critical Care Medicine, University of Pisa, Pisa, Italy; 7 Translational Medicine Department, Eastern Piedmont University, Novara, Italy; 8 Anesthesia and Intensive Care, “Maggiore Della Carità” Hospital, Novara, Italy; 9 Department of Clinical and Experimental Medicine, University of Catania, Catania, Italy; 10 Anesthesiology, Critical Care and Pain Medicine Division, Department of Medicine and Surgery, University of Parma, Parma, Italy; 11 Ospedale Uboldo, Cernusco sul Naviglio, Milan, Italy

**Keywords:** Diaphragm ultrasound, Diaphragm imaging, Learning, Education, Course, Training, Critical care, Intensive care unit

## Abstract

**Background:**

This study aims to ascertain whether (1) an educational program is sufficient to achieve adequate Diaphragm Ultrasound (DUS) assessments on healthy volunteers and (2) combining a video tutorial with a practical session is more effective in making learners capable to obtain accurate DUS measurements, as opposed to sole video tutorial.

**Results:**

We enrolledstep 1: 172 volunteers naïve to ultrasound. After watching a video tutorial, a questionnaire was administered and considered to be passed when at least 70% of the questions were correctly answered. Course participants who passed the theoretical test were randomized to either intervention or control group. Learners randomized to the interventional group underwent to a practical training, tutored by an expert, before accessing DUS examination. Participants randomized to the control group directly accessed DUS examination, without any practical training. DUS measurements by learners and tutors were recorded and checked for accuracy, according to predefined criteria. Detection of both acoustic windows and accurate DUS assessment was achieved by 83.7% learners of the intervention group while 3.5% only among controls (*p* < 0.0001). The subcostal view of the diaphragm was correctly identified by 92% and 65% learners in the intervention and control groups, respectively (*p* < 0.0001) while the apposition zone by 86% and 71% learners, respectively (*p* = 0.026). An accurate diaphragm displacement (DD) measurement was obtained by 91% and 45% learners in the intervention and control groups, respectively (*p* < 0.0001) while an accurate thickening fraction (TF) measurement by 99% and 21%, respectively (*p* < 0.0001). DD measurements by both groups of learners were significantly correlated with those assessed by expert tutors; however, a significant improvement of measurement accuracy was found in learners randomized to receive also the practical training, compared to controls.

**Conclusions:**

A combined approach consisting of a theoretical module followed by a practical training is more effective in managing acoustic windows and performing accurate measurements when compared to an exclusively theoretical course.

*Trial registration* prospectively registered on clinicaltrials.gov (Identifier: NCT03704129; release date 17th October 2018).

## Background

Diaphragm ultrasonography (DUS) allows serial radiation-free bedside evaluations of diaphragmatic function in critically ill patients [1, 2]. DUS allows the assessment of both cranio-caudal diaphragm displacement (DD) from subcostal acoustic windows, and thickness at the end of inspiration (Thick_insp_) and expiration (Thick_exp_) in the zone of apposition, to compute the thickening fraction (TF) as Thick_insp_ − Thick_exp_/Thick_exp_ [1].

While some studies evaluated the educational approaches for the achievement of skills for specific cardiac and lung ultrasound assessments, no study has so far evaluated how to develop appropriate DUS skills, despite the increased interest for this technique [3, 4]. Though a fair amount of tutored examinations is necessary to achieve appropriate skills for patient assessment by ultrasonography [5], basic knowledge is necessary before starting evaluating patients.

The present study aims to ascertain whether (1) a brief educational program is sufficient to achieve adequate DUS assessments on healthy volunteers and (2) combining a video tutorial with a practical session on healthy volunteers is more effective in making learners with no previous experience capable to obtain accurate DUS measurements, as opposed to the sole video tutorial. We, therefore, compared these two educational approaches and assessed the rates of learners able to correctly detect the two acoustic windows (subcostal view and apposition zone), and the correlation between measurements (DD, Thick_insp_, Thick_exp,_ and TF) performed by learners and tutors.

## Materials and methods

The study was carried out from December 1st 2018 to February 28th 2019 in the educational rooms of eight Italian University Hospitals (“Magna Graecia” University of Catanzaro, IRCCS San Raffaele Scientific Institute of Milan, University of Udine, “Eastern Piedmont” University of Novara, University of Pisa, University of Parma, University of Catania and Catholic University of the “Sacred Heart” of Rome). The study was approved by the local Ethics Committees and written informed consent was obtained from all participants. The trial was prospectively registered on clinicaltrials.gov (Identifier: NCT03704129; release date 17th October 2018).

### Subjects

We recruited 172 voluntary learners with no experience of ultrasound assessments among medical students or first-year residents. We also designated 14 tutors, two in each centre, with a minimum 2-year experience of DUS in critical care US. Prior to study initiation, all tutors met on the web to standardize the practical training to be administered to the interventional group.

### Study protocol

A video tutorial based on the current literature [1, 6–9] and focusing on key principles of the technique, including acoustic windows and anatomical landmarks featuring diaphragmatic US, was shown to all learners. The video tutorial is available online at https://youtu.be/B7AYP9fElyE.

Afterwards, a questionnaire including 10 multiple-choice questions was administered and considered to be passed when at least 70% of the questions were correctly answered. The questionnaire is enclosed as Additional file 1. Course participants who passed the theoretical test were then randomized to either intervention or control group.

Randomization was achieved with an allocation ratio of 1:1 by means of a computer-generated sequence, operated by an investigator not involved in the trial. Allocation blindness was assured using sequentially numbered sealed opaque envelopes, prepared by the aforementioned investigator. Each envelope contained the allocation of the learners to either control or interventional group, with a unique identifier code. The randomization was based on a centralized phone call system.

Learners randomized to the interventional group had access to the practical training, tutored by an expert evaluator who interactively explained how to perform DUS, before accessing DUS examination. Learners randomized to the control group directly accessed DUS examination, without any practical training by expert tutors. DUS examination was performed on healthy volunteers, not involved in the study protocol. Irrespective of the group of randomizations, learners were asked to independently perform DUS using both acoustic windows. All measurements were performed by learners after images’ acquisition and storage. A local investigator recorded the measurements. A tutor then judged if the images were correctly acquired, and only in such a case, he performed his own measurements on the same acquired images, being blind to the results obtained by the learners. These measurements were also recorded by the local investigator.

### Data acquisition and analysis

Diaphragm US was performed by course participants and tutors using one of the following devices: MyLab™30, Esaote, Genova, Italy; MySono U6, Samsung, Seoul, South Korea; EPIQ7 ultrasound system, Philips Healthcare, Bothell, WA, USA.

Sonographic evaluation was conducted on the right hemi-diaphragm, as previously described [1, 6, 9–11]. Briefly, DD was ascertained through a 3.5–5 MHz phased array probe, placed immediately below the costal margin in the mid-clavicular line and directed medially, cephalad and dorsally, so that the US beam reached perpendicularly the posterior third of the hemi-diaphragm [1, 6, 9, 11]. The motion of the diaphragm and other anatomical structures along the selected line was displayed in “time-motion” mode (M-mode). DD was measured placing the first caliper at the end of expiration phase, while the second caliper was placed at the apex of inspiration slope [1, 6, 9, 11]. Diaphragm thickness was assessed through a linear 13 MHz probe placed in the 9th–10th intercostal space, closed to the midaxillary line, angled perpendicular to the chest wall, to identify the apposition zone of the diaphragm. Diaphragmatic thickness was the determined in M-mode at end-expiration (Thick_exp_) and at peak inspiration (Thick_insp_) as the distance between the diaphragmatic pleura and the peritoneum [6, 7, 9]. TF was then computed as Thick_insp_ − Thick_exp_/Thick_exp_ and expressed in percentage [6, 7, 9].

If a learner failed to correctly display the diaphragm in one of the two acoustic windows, the examination was considered to be negative. Only the measurements by learners who correctly identified both acoustic windows were considered for further analysis. Based on the previous agreement among members of the steering committee, the measurements were considered to be accurate when in the following predetermined ranges: (1) DD ± 2 mm from the value reported by the tutor and (2) TF ± 20% of the assessment recorded by the tutor. If both acoustic windows were correctly identified, and the resulting measurements were included within the predetermined ranges, the participant passed the examination, so that the first study outcome was achieved.

### Statistical analysis

Gaussian data distribution was tested by means of the Kolmogorov–Smirnov test. Data are presented as mean (± standard deviation) or as median [25th–75th interquartile], as indicated. Categorical data were compared through Chi-square test while continuous data with Student *t* test or Mann–Whitney *U* test, as appropriate. By means of the Spearman’s rank correlation test, we determined the correlation coefficients (*ρ*) [95% interval confidence] between measurements (i.e., DD, Thick_insp_, Thick_exp,_ and TF) obtained by tutors and learners of each group, and the corresponding *p* values. To test the statistical significance of the difference, the *ρ* values separately obtained in the intervention and control groups were then compared and the *z* and *p* values were determined [12]. For all comparisons, *p* values < 0.05 were considered to be significant.

## Results

All 172 learners passed the multiple-choice questionnaire test (score 95.5 ± 7.6%) accessing to the second phase of the study, and were randomized to either intervention or control group (86 subjects in both groups). Neither age nor female/male ratio were different between groups.

**Fig. 1:**
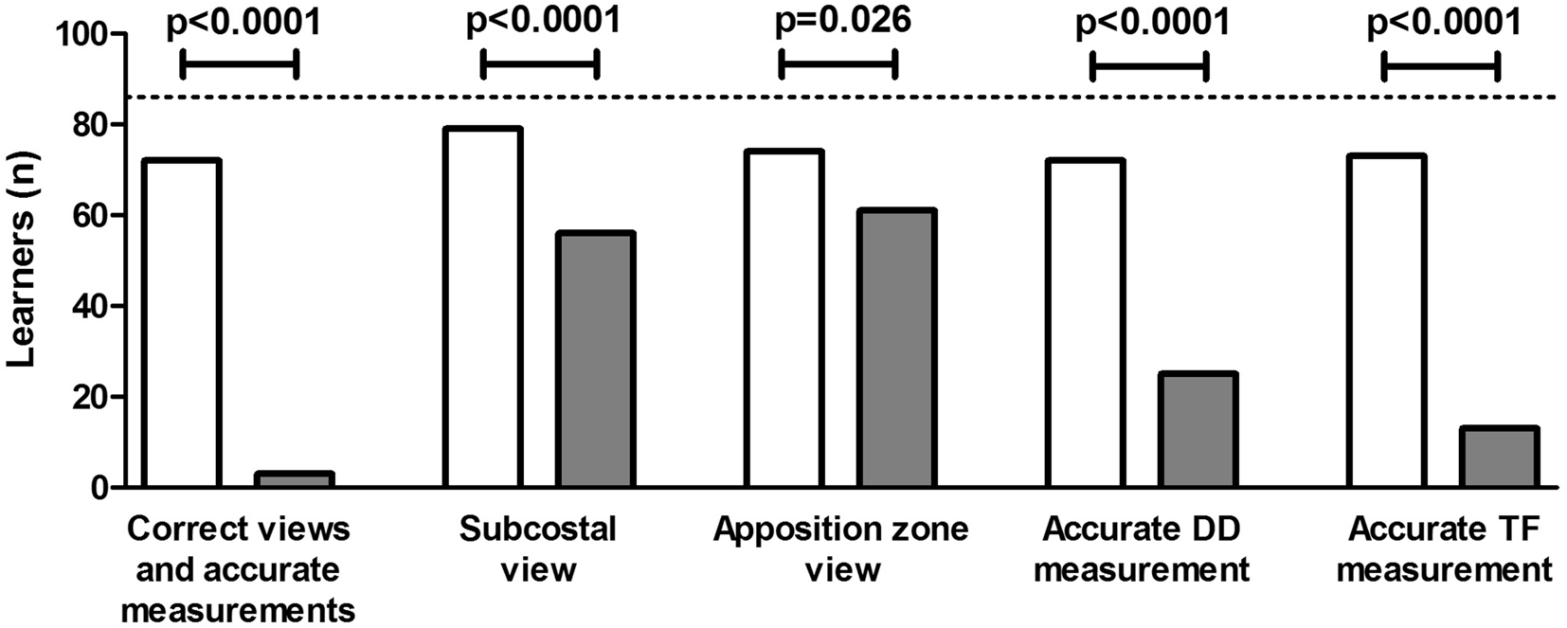
Number of learners reaching the study aims. From left to the right, the figure depicts the number of learners that: (1) correctly detected both acoustic windows and performed accurate measurements (first outcome); (2) correctly identified the subcostal view; (3) correctly identified the apposition zone; (4) accurately performed the DD measurement; and (5) accurately performed the TF measurement. White bars represent the intervention group while grey ones the control group. The dashed line represents the total number of learners randomized per group (*n* = 86)

As depicted in Fig. 1, correct detection of both acoustic windows and accurate measurements was overall obtained by 72/86 (83.7%) learners allocated in the intervention (white bar) group, as opposed to 3/86 (3.5%) in the control group (grey bar) (*p* < 0.0001). The subcostal view of the diaphragm was correctly identified by 79/86 (92%) and 56/86 (65%) learners in the intervention and control groups, respectively (*p* < 0.0001) while the apposition zone of the diaphragm by 74/86 (86%) and 61/86 (71%) learners in the intervention and control groups, respectively (*p* = 0.026). An accurate DD measurement was obtained by 72/79 (91%) and 25/56 (45%) learners in the intervention and control groups, respectively (*p* < 0.0001) while an accurate TF measurement by 73/74 (99%) and 13/61 (21%) learners in the intervention and control groups, respectively (*p* < 0.0001) (see Fig. 1).

Table 1 displays correlations of the measurements done by tutors and learners in the two groups. DD measurements by both groups of learners were significantly correlated with those assessed by expert tutors; however, a significant improvement of measurement accuracy was found in learners randomized to receive also the practical training, compared to controls. The measurements of Thick_insp_, Thick_exp,_ and TF performed by learners randomized in the intervention group were found to be strongly correlated with those obtained by the tutors, in contrast to those obtained by learners in the control group, as also indicated by the significant differences of the correlation comparisons.

**Table 1:** Correlation between tutors and learners for each DUS parameter and correlation comparison between groups

Parameter	Intervention group			Control group			Correlation comparison	
	Learners (*n*)	*ρ* [95% CI]	*p* value	Learners (n)	*ρ* [95% CI]	*p* value	*z* value	*p* value
DD	80	0.973 [0.958–0.983]	< 0.0001	50	0.802 [0.675–0.883]	< 0.0001	− 5627	< 0.0001
Thick_insp_	81	0.949 [0.921–0.976]	< 0.0001	58	0.209 [− 0.052–0.444]	0.115	− 9.141	< 0.0001
Thick_exp_	81	0.938 [0.905–0.960]	< 0.0001	58	0.310 [0.057–0.526]	0.018	− 7.955	< 0.0001
TF	81	0.975 [0.961–0.960]	< 0.0001	58	− 0.028 [− 0.284–0.232]	0.836	− 12.061	< 0.0001

DUS, diaphragm ultrasound; *ρ*, correlation coefficient; 95% CI, 95% confidence interval; DD, diaphragm displacement; Thick_insp_, diaphragm thickness at inspiration; Thick_exp_, diaphragm thickness at expiration; TF, thickening fraction

## Discussion

Our results show that (1) a brief educational program can make learners naive to the technique able to perform DUS on healthy volunteers; (2) compared to the sole theoretical teaching, the addition of a brief practical training improves (i) the ability to correctly detect the two acoustic windows and to perform accurate diaphragm US measurements; (ii) the rate of correct identifications of the two acoustic windows of the diaphragm; and (iii) the accuracy of DUS measurements.

To our knowledge, this is the first study evaluating a DUS educational program. Some studies evaluated educational programs for other applications of bedside ultrasound. Beaulieu et al., an initial web-based theoretical program followed by hands-on training performed by simulation and on both healthy and sick individuals, improved the ability of 37 junior emergency medicine residents to recognize both venous vessels (5.5 h) and pleura (2.5 h) [13]. Lim et al. showed that a 3-h educational course of lung ultrasound, combining 1 h of theory and 2 h of hands-on training on healthy subjects, significantly improved 40 medical students’ knowledge, image acquisition, and interpretation [14]. Melamed et al. administered a 2-h theoretical instruction on transthoracic echocardiography followed by a 4-h hands’ training on critically ill patients with (20 patients) or without (24 patients) left ventricular abnormalities, to previously untrained ICU physicians [15]. Afterwards, they evaluated their ability to detect normal or altered left ventricular function and found a proper detection in 86% of the cases. None of these studies, however, did not evaluate the importance of adding a practical training session, compared to the sole theory [13–15].

The accuracy of DD measurement was overall fairly reliable. However, in the control group, the rate of learners who properly identified through the subcostal acoustic window the hemidiaphragm was lower compared with the corrected detection of the hemidiaphragm thickness in the zone of apposition, 58% vs. 67%. Indeed, a poor acoustic window may occur in up to 10% of cases [16]. Moreover, the identification of the right hemidiaphragm through the subcostal acoustic window requires recognizing the anatomy of the liver, which is strongly dependent on the ability of the operator to direct the ultrasound beam cephalically and dorsally to this organ to reach the hemidiaphragm perpendicularly [17]. Quite the opposite, with the high-frequency and low-penetrance linear probe used to identify the diaphragm zone of apposition, the thickness is easily identified being located just below the skin [18]. However, measuring Thick_insp_ and Thick_exp_ is much more difficult than assessing DD because of the small dimensions, which also makes the M-mode image hard to measure. Small errors in Thick_insp_ and Thick_exp_ determination may consequently affect TF computation. That said, it is quite striking that learners in the intervention group were able to achieve measurements quite close to those obtained by the tutors.

Compared to the previous studies evaluating training on ultrasound for lung, heart, and venous vessels bedside evaluation [13–15], our study has the point of strength of enrolling a high number of trainees on a multicenter basis.

The major limitation of this study, that we share with the previous analogous investigations [13, 14, 19], is that learners performed DUS in healthy volunteers, which does not imply they would successfully perform unattended DUS evaluation in the clinical arena, where potential confounding factors such as underlying disease, mechanical ventilation, or abdominal distension can make DUS assessment more problematic. Rouby et al. demonstrated that 25 lung ultrasound examinations supervised by an expert tutor are enough to acquire adequate technical skills in critically ill patients [5]. Bergamaschi et al. also showed that a 3-h course followed by 6-h practical training and 25 supervised examinations was enough to acquire the ability to determine cardiac output by trans-thoracic echocardiography [20]. Data for DUS at this regard lack, but we believe that 25 supervised examinations would be sufficient to achieve adequate DUS skills for critically ill patients’ examination.

## Conclusions

DUS can be easily taught to naïve to ultrasounds students. A combined approach consisting of a theoretical module followed by a practical training is more effective in managing acoustic windows and performing accurate measurements when compared to an exclusively theoretical session. After theoretical and practical training, the vast majority of learners accurately explored the diaphragm, thus obtaining DUS results consistent with those detected by an expert tutor.

## Supplementary information

**Additional file 1.** Theoretical test.

## Data Availability

The authors will share all of the individual participant data collected during the trial after deidentification, to researchers who provide a methodologically sound proposal. For data availability contact: longhini.federico@gmail.com

## Abbreviations

DD: diaphragm displacement
DUS: diaphragm ultrasound
TF: thickening fraction
Thick_insp_: thickness at the end of inspiration
Thick_exp_: thickness at the end of expiration
