# Supplementary material for: Comparisons of two diaphragm ultrasound-teaching programs: a multicenter randomized controlled educational study
Source: Ultrasound J. 2019 Oct 3;11:21. doi: 10.1186/s13089-019-0137-4 (PMC6775177; doi:10.1186/s13089-019-0137-4)
Supplement: Supplementary file 1 — Additional file 1. Theoretical test. [file 13089_2019_137_MOESM1_ESM.docx]

**Additional Material**

**COMPARISONS OF TWO DIAPHRAGM ULTRASOUND TEACHING PROGRAMS: A MULTICENTER RANDOMIZED CONTROLLED EDUCATIONAL STUDY.**

Eugenio Garofalo; Andrea Bruni; Corrado Pelaia; Giovanni Landoni; Alberto Zangrillo; Massimo Antonelli; Giorgio Conti; Daniele Guerino Biasucci; Giovanna Mercurio; Andrea Cortegiani; Antonino Giarratano; Luigi Vetrugno; Tiziana Bove; Francesco Forfori; Francesco Corradi; Rosanna Vaschetto; Gianmaria Cammarota; Marinella Astuto; Paolo Murabito; Valentina Bellini; Massimo Zambon; Federico Longhini; Paolo Navalesi; Elena Bignami.

**THEORETICAL TEST**

1. On which physical principle diagnostic imaging by ultrasound is based?
   1. Electromagnetic radiation
   2. **Mechanical waves**
   3. Positron emission
   4. X-rays
   5. Gamma-rays
2. What ultrasound frequency range is used?
   1. 1-10 kHz
   2. 2-20 kHz
   3. 1-10 MHz
   4. **2-20 MHz**
   5. 20-200 MHz
3. How a hypoechogenic structure appears with respect to the surrounding structures?
   1. **It reflects fewer echoes**
   2. It reflects more echoes
   3. It does not reflect echoes
   4. It looks brighter
   5. It appears completely black
4. A linear probe is characterized by:
   1. High penetration
   2. Low spatial resolution
   3. Low frequency
   4. **High frequency**
   5. Good visualization of deep structures
5. What sentence is true about the M-Mode?:
   1. It is static
   2. **It evaluates the movement of a structure**
   3. It is never used in echocardiography
   4. It uses the same parameters of B-mode
   5. It is three-dimensional
6. The normal value of the diaphragmatic inspiratory displacement is about:
   1. 0.34 cm
   2. **1.34 cm**
   3. 2.34 cm
   4. 3.34 cm
   5. 4.34 cm
7. The normal value of the diaphragm thickening fraction is about:
   1. 0-10%
   2. 10-20%
   3. **30-40%**
   4. 50-60%
   5. 70-80%
8. At level of the apposition zone:
   1. A convex probe (2-5 MHz) is used
   2. The probe is placed in the subcostal area
   3. The displacement of the diaphragm is evaluated
   4. The hepatic window is used
   5. **The diaphragm thickening is evaluated**
9. In B-mode, at level of the apposition zone, the diaphragm appears as:
   1. An anechogenic track between two hypoechogenic lines
   2. A hyperechogenic line
   3. **An anechogenic track between two hyperechogenic lines**
   4. An artifact
   5. A shadow cone

10. The diaphragm thickening fraction (TF) is calculated by:

- 1. **TF=(Thickness at the end of inspiration-Thickness at the end of expiration)/Thickness at the end of expiration**
  2. TF=Thickness at the end of inspiration/Thickness at the end of expiration
  3. TF=(Thickness at the end of expiration-Thickness at the end of inspiration)/Thickness at the end of inspiration
  4. TF=Thickness at the end of expiration/Thickness at the end of inspiration
  5. It cannot be calculated
